# Migration health ethics in Southeast Asia: a scoping review

**DOI:** 10.12688/wellcomeopenres.19572.2

**Published:** 2023-11-20

**Authors:** Shu Hui Ng, Sharon Kaur, Phaik Yeong Cheah, Zhen Ling Ong, Jane Lim, Teck Chuan Voo

**Affiliations:** 1 School of Medicine and Health Sciences, Monash University Malaysia, Bandar Sunway, Selangor, 47500, Malaysia; 2 Faculty of Law, University Malaya, Kuala Lumpur, Federal Territory of Kuala Lumpur, 50603, Malaysia; 3 Mahidol Oxford Tropical Medicine Research Unit, Bangkok, 10400, Thailand; 4 Centre for Tropical Medicine and Global Health, Oxford, OX3 7LG, UK; 5 Centre for Epidemiology and Evidence-based Practice, Department of Social and Preventive Medicine, University Malaya, Kuala Lumpur, Federal Territory of Kuala Lumpur, 50603, Malaysia; 6 Department of Global Health and Development, Faculty of Public Health and Policy, London School of Hygiene and Tropical Medicine, London, WC1E 7HT, UK; 7 Centre for Biomedical Ethics, Yong Loo Lin School of Medicine, National University of Singapore, Singapore, 117597, Singapore

**Keywords:** Bioethics, Southeast Asia, Research Ethics, Clinical Ethics, Public Health Ethics, Migration Health, Migrants, Refugees

## Abstract

**Background:**

Asia hosts the second-largest international migrant population in the world. In Southeast Asia (SEA), key types of migration are labour migration, forced migration, and environmental migration. This scoping review seeks to identify key themes and gaps in current research on the ethics of healthcare for mobile and marginalised populations in SEA, and the ethics of research involving these populations.

**Methods:**

We performed a scoping review using three broad concepts: population (stateless population, migrants, refugees, asylum seekers, internally displaced people), issues (healthcare and ethics), and context (11 countries in SEA). Three databases (PubMed, CINAHL, and Web of Science) were searched from 2000 until May 2023 over a period of four months (February 2023 to May 2023). Other relevant publications were identified through citation searches, and six bioethics journals were hand searched. All searches were conducted in English, and relevant publications were screened against the inclusion and exclusion criteria. Data were subsequently imported into NVivo 14, and thematic analysis was conducted.

**Results:**

We identified 18 papers with substantial bioethical analysis. Ethical concepts that guide the analysis were ‘capability, agency, dignity’, ‘vulnerability’, ‘precarity, complicity, and structural violence’ (n=7). Ethical issues were discussed from the perspective of research ethics (n=9), clinical ethics (n=1) and public health ethics (n=1). All publications are from researchers based in Singapore, Thailand, and Malaysia. Research gaps identified include the need for more research involving migrant children, research from migrant-sending countries, studies on quality of migrant healthcare, participatory health research, and research with internal migrants.

**Conclusions:**

More empirical research is necessary to better understand the ethical issues that exist in the domains of research, clinical care, and public health. Critical examination of the interplay between migration, health and ethics with consideration of the diverse factors and contexts involved is crucial for the advancement of migration health ethics in SEA.

## Introduction

This scoping review by the Southeast Asia Bioethics Network, ^1^ seeks to identify research gaps in the ethics of healthcare for and research with marginalised migrant populations within Southeast Asia (SEA). The first phase of this research is to identify key themes in bioethics-related research within the SEA region, and thus serve as a reference to those with an interest in the ethics of migration health, including but not limited to researchers, policymakers, healthcare practitioners, and members or representatives of migrant communities. For the purpose of this paper, the word ‘migrant’ will include asylum seekers, refugees, internally displaced persons, stateless persons, and economic or labour migrants in regular or irregular situations, but exclude professional migrants, marriage migrants, international students and tourists. While we acknowledge that there are important ethical considerations related to the migration and health of the excluded migrant groups, it is beyond the scope of this paper to address these issues.

This paper begins with a brief overview of the migration landscape in SEA, with focus on the main reasons prompting migration in this region, followed by a discussion on why migration is a complex determinant of health. It then provides an outline of the role of bioethics in advancing migration health. Then, using a scoping review methodology, literature related to the ethics of migration health was analysed thematically and presented in the results section. Engagement with regional stakeholders of migration health was also undertaken to attain a more holistic overview of the research priorities in advancing regional migration health. In the discussion section, the value of conceptual and empirical bioethics research is discussed, and key research gaps identified by the scoping review are highlighted. The paper concludes by summarising the potential roles of the SEA Bioethics Network in advancing migration health ethics in this region.

### Migration: A Southeast Asian context

The term ‘migrant’ is an umbrella term not defined under international law but is defined by the International Organization for Migration (IOM) as “a person who moves away from his or her place of usual residence, whether within a country or across an international border, temporarily or permanently, and for a variety of reasons” ( Sironi *et al.,* 2019, p.132). Over the span of the last few decades, the number of migrants has increased exponentially across the globe ( McAuliffe & Triandafyllidou, 2021). Due to an intensification of intercontinental connectivity and mobility, the IOM estimates that there were around 281 million international migrants worldwide in 2020, which equates to 3.6% of the global population ( McAuliffe & Triandafyllidou, 2021). Europe with 87 million migrants (30.9% of the international migrant population) is currently the largest destination for international migrants, followed closely by the 86 million international migrants living in Asia (30.5%) ( McAuliffe & Triandafyllidou, 2021). It is worth noting that the majority of migration takes place within countries and not across international borders, making the actual global migrant population larger than estimated. The latest data in 2009 recorded 740 million internal migrants ( Esmer *et al.*, 2009).

The SEA which includes 11 countries, i.e., Lao People’s Democratic Republic (PDR), Vietnam, Cambodia, Myanmar, Thailand, Malaysia, Singapore, Brunei, Philippines, Timor-Leste, and Indonesia, has a long history of population movement ( Migration Data Portal, 2022). With bilateral agreements and national labour-export programs, labour migration continued to increase over the years. Forced migration movements are also significant, with over a million Rohingyas fleeing Myanmar in successive waves since the early 1990s. Today, migration in SEA continues to increase due to conflict, enhanced mobility infrastructure, and employment-related issues. While North America, Europe and Western Asia are important regions of destination, the majority of the 23.6 million migrants from SEA do not migrate out of the region ( Migration Data Portal, 2022).

Owing to disparate socioeconomic development, civil unrest and natural disasters, many from the SEA region migrate in search of better living conditions and livelihood opportunities. Besides that, irregular migration, including the smuggling and trafficking of persons, is also an issue of regional importance ( IOM Thailand, n.d.). The key drivers for migration in SEA can be broadly categorised into labour migration, forced migration driven by regional conflict and violence, and environmental migration.

***i) Labour migration***

Regional economic disparities within and amongst SEA countries have led to a high number of labour migrants, and has resulted in the region experiencing one of the highest rates of population mobility in the world ( König *et al.,* 2022). “According to the World Bank, the Association of Southeast Asian Nations (ASEAN) region accounts for 8% of the world’s migrants and hosts 4% of the world’s migrants”, with the majority of migrant workers working in lower-skilled occupations in the region ( ASEAN Secretariat, 2022). Cambodia, Indonesia, Lao PDR, Myanmar, the Philippines, and Vietnam are net-sending countries, while Brunei, Malaysia, Singapore, and Thailand are net-receiving countries of labour migration ( Pasadilla, 2011).

As of December 2022, the foreign workforce in Singapore numbered 1.4 million ( Ministry of Manpower Singapore, n.d.). Thailand, a major migrant-receiving country, has 2.49 million documented migrant workers as of March 2023 ( ILO Triangle in ASEAN, 2023). Conversely, the Philippines has nearly 11% of its total population living or working outside of the country, a value followed closely by Vietnam ( McAuliffe & Triandafyllidou, 2021). Vietnam is predominantly a migrant-sending nation, but recent census data shows an increasing trend of internal rural-urban migration ( IOM, 2020). Rural poverty, decline in agriculture and increased demand for labour in urban centers has resulted in significant internal rural-urban migration in SEA, particularly in countries such as Cambodia, Timor-Leste, and Vietnam ( UNESCO, 2018). Notwithstanding formal labour migration, there are irregular migrant workers in SEA who also contribute to the labour workforce, but this data is not reflected in official statistics ( ILO Regional Office for Asia and Pacific, 2022).

Conventionally, labour migration benefits migrant-receiving countries as it helps catalyse economic growth dependent on foreign labour, while migrant-sending countries stand to gain from a reduction in the rate of unemployment and increased income via foreign remittances ( Elmhirst, 2013). Nonetheless, it has been documented that many labour migrants particularly those in informal sectors are exposed to a multitude of abuses and mistreatment, including but not limited to forced labour ( McAuliffe & Triandafyllidou, 2021). Recent figures from the International Labour Organisation (ILO) indicated that more than 11 million people in Asia Pacific are victims of forced labour, ^2^ accounting for well over half of the global estimated number of 21 million victims, most of whom are involved in the sectors of agriculture, construction, manufacturing, entertainment and domestic work ( ILO, 2018).

***ii) Forced migration driven by conflict and violence***

The Rohingya people are the world’s largest stateless population, a circumstance contributed in part by their systematic exclusion from Myanmar citizenship since 1982 ( USA for UNHCR, n.d.). Being stateless, the Rohingya are denied basic rights and are exposed to various forms of abuse and exploitation ( UNHCR, 2022). Heightened violence against the Rohingyas in Rakhine State in 2017 and the subsequent military coup of February 2021 have caused massive displacements and the forced migration of the Rohingya regionally and internationally. As a result of the February 2021 military takeover, more than one million remained internally displaced from their homes, and at least 70,000 have fled Myanmar, adding to millions of Myanmar migrants with irregular status in the region ( McAuliffe & Triandafyllidou, 2021). As of December 2022, the refugee camps in Bangladesh’s Cox’s Bazaar are reported to host close to one million Rohingyas ( UNHCR Operational Data Portal, n.d.). Overcrowding, poor sanitation ( English, 2018), devastating fires ( de la Portilla, 2022), heavy rain and landslides ( Relief Web, 2021), and violent criminal activities ( Aziz, 2022) have all given rise to increasingly dire humanitarian crises in refugee camps, pushing the Rohingyas into a constant state of forced displacement. Data from UNHCR in January 2023 revealed a 360% increase in Rohingya refugees attempting perilous sea journeys in search of protection, security, family reunification, and improved livelihoods in other countries within SEA ( UNHCR Flash Update, 2023). 

The issue of forced migration is not limited to the Rohingya. SEA is also host to refugees and asylum seekers from other parts of the world, including but not limited to Pakistan, Yemen, Afghanistan, Palestine, Somalia and Iraq. At the time of writing, there are significant numbers of refugees and asylum seekers in Malaysia (183,790 registered refugees, with a majority of them from Myanmar) ( UNHCR Malaysia, 2023), Thailand (90,630 in refugee camps, 5000 urban refugees, 480,00 stateless persons) ( UNHCR Thailand, 2023), followed by Indonesia (12,706 persons of concern) ( UNHCR Indonesia, 2023).

***iii) Environmental migration***

The IOM defines environmental migrants as “persons or groups of persons who, for compelling reasons of sudden or progressive changes in the environment that adversely affect their lives or living conditions, are obliged to leave their habitual homes, or choose to do so, either temporarily or permanently, and who move either within their country or abroad” ( Kälin & Weerasinghe, 2017, p. 1) SEA, which is home to more than 640 million people, is vulnerable to extreme weather events and rising sea levels associated with climate change ( Prakash, 2018). In 2020, as a result of the Mount Taal volcanic eruption, cyclones, storms and flooding, the Philippines, Vietnam and Indonesia recorded more than six million displacements combined ( McAuliffe & Triandafyllidou, 2021). Periodic major environmental disasters aside, in the Mekong Delta, seasonal migration towards urban centres for work during the high flood season is a common practice, especially amongst rice farmers dependent on agriculture for livelihood ( Dun, 2011). Similarly, Timor-Leste's geographical attributes mean that it is also particularly susceptible to natural hazards, such as droughts, floods and landslides ( IOM Timor-Leste, n.d.).

Beyond these three major drivers of migration, there are also other diverse reasons for migration within and across countries ( Migration Data Portal, 2022). Migration is a global reality, and although the majority of global migration occurs within low- and middle-income countries (LMICs), migration from LMICs to high-income countries (HICs) forms the most prominent dialogue ( Abubakar *et al.,* 2018). The discourse on migration is dominated by debates on immigration and border control, with gaps in understanding the relationship between migration and health. The health of migrants remains at the margins of policy making in countries at all income levels, and responses have generally revolved around disease control programmes such as immigration health assessments, health screening for fitness to work or travel, and border quarantine ( Wickramage *et al.,* 2019).

### Migration as a complex determinant of health

Health is defined as “a state of complete physical, mental and social well-being” ( WHO, 1948). Migration is a complex multi-directional process: it can be short-term, permanent, circular or result in a return to the country of origin, and it can be regular or irregular ( ASEAN Migration Outlook, 2022). At different phases of migration, migrants’ interactions with determinants of health will determine their health before, during or after their migration journey. For example, migration can potentially lead to poorer health due to precarious working conditions and poor access to healthcare or improve health by reducing the risks of encountering violence in the home country or by having better healthcare access in destination countries ( Vearey *et al.,* 2020). Table 1 summarises the determinants of health for migrants at different phases of migration.

**Table 1.** Determinants of health at different phases of migration ( Legido-Quigley *et al.,* 2020; Vearey *et al.,* 2020).

Pre-migration phase
▪ Genetic and biological characteristics. ▪ Epidemiological profiles at origin (e.g. endemicity, infectious and chronic disease patterns). ▪ Exposures to trauma from protracted conflicts, human rights violations and interpersonal violence. ▪ Baseline health status, access to basic health services and nutrition in the country of origin.
Movement phase
▪ Duration, circumstances and condition of journey. Overcrowded and unsafe living conditions, inadequate shelter, poor sanitation, insufficient food and water. ▪ Experiencing or witnessing violence, exploitation and other abuses.
Interception phase
▪ Injuries due to abuse, sexual violence and difficult journey. ▪ Starvation and nutritional deficits. ▪ Mental trauma. ▪ Infectious diseases and untreated chronic conditions.
Arrival and integration phase
▪ Domestic migration policies and legal framework govern migrants’ access to health services (based on their legal status). Barriers to health services including financial, legal, structural, physical and social determinants. ▪ Overcrowded living conditions upon arrival leading to various skin and respiratory infections. ▪ Limited health services in detention centres. ▪ Health risks behaviours and vulnerabilities among migrants and families. ▪ Language and cultural values - linguistically and culturally sensitive health service provision. ▪ Racism, social exclusion, discrimination, exploitation. ▪ Family/partner separation and stress.
Return/Resettlement
▪ Duration of absence. ▪ Reintegration with family, household, and community at origin. ▪ Migrants may return to households that have benefitted from remittance flows and promote positive health trajectories, or maybe worse-off physically and psychologically with the cumulative tolls of their migration journeys. ▪ Delay in establishing/seeking healthcare as a result of long resettlement process.

The UN 2030 Agenda for Sustainable Development acknowledges that health is a fundamental precondition for migrants to work productively and contribute to the development of communities of origin and destination, and thus coordinated efforts are needed to ensure that the health of migrants is addressed throughout all migration phases ( IOM Migration Health Division, n.d.). Migration is thus a complex determinant of health with dynamic interactions with legal, social, economic and public health considerations.

### The role of bioethics in SEA migration health

Bioethics is concerned with ethical issues relating to health and life sciences ( Dawson *et al.,* 2018). It is an “interdisciplinary field populated by scholars, teachers, and clinical practitioners from a wide variety of traditional disciplines, such as philosophy, religious studies, law, medicine, nursing, social work, public health, the medical humanities (literature and history), and social sciences (politics, sociology, economics, anthropology)” ( University of Virginia, 2023). Onarheim *et al.* (2021) highlighted myriad ethical dilemmas faced by key stakeholders in migration health, which include health workers, regional and international policymakers, data managers and researchers, and migrants themselves, which suggests that bioethics is inextricably linked to almost all aspects of migration health.

### Healthcare Access

In terms of migrants’ healthcare access, various ethical normative questions can arise - questions such as “To what extent is the responsibility of receiving countries in providing healthcare for migrants?” “Are policies limiting healthcare to migrants morally acceptable?”, and “Should there be a difference in healthcare coverage for different groups of migrants?” ( Wild, 2012). In the context of resource-limited settings, is it ethically justifiable for (undocumented) migrants to receive similar health coverage as citizens? These questions could, and should be analysed through contextualised ethical lenses, to advocate for policies and practices that support appropriate and equal healthcare access for migrants.

### Research Ethics

While ethical concerns are pertinent for all research participants, research involving migrant populations produces a more complex set of ethical conundrums ( Parker, 2012). Research with migrants requires special consideration of issues surrounding potential exploitation, informed consent, and respect for participant autonomy ( Lott, 2005). The necessity and utility of research with marginalised migrant populations should be thoroughly assessed to ensure that the research seeks to provide new information that is of social value to the study population ( Global Forum of Bioethics in Research Meeting Report, 2017). Migration health researchers should also be cognisant of the fact that power inequalities experienced in research can leave specific members of a society (including migrants) dependent on the decisions of others, and that ethics of research is not limited to ethics board approval ( Pottie & Gabriel, 2014).

One important way in which bioethics can contribute to migration health is through advocacy research ^3^, especially on behalf of others who are marginalised and systematically deprived of the ability to act in their own interests ( Dawson *et al.,* 2018). Research in bioethics hold a unique position to analyse ways in which migrants’ right to health is undermined and ways to redress breaches of human rights and equity ( Zion, 2019). Research on migration health ethics can also be transformative – bringing disempowered voices into focus and providing a platform that offers solutions to pertinent issues surrounding migrant health ( Zion, 2019). It is imperative to not only recognise ethical challenges in migration health, but also to document the issues while systematically formulating sustainable ethical solutions to research, clinical practice and policymaking on migration health ( Onarheim *et al.,* 2021).

## Methods

### Objectives

Despite the burgeoning academic literature on migration health in SEA, our hypothesis is that there is limited migration-health related normative bioethical analysis (e.g., literature prescribing what practitioners ought to do) and empirical bioethics research (e.g., literature describing what is the ‘experiential landscape’ in which ethical decisions and practices occur, pertaining to current opinions, values and practices etc) ( Kon, 2009; Rehmann-sutter *et al.*, 2012). We also hypothesise that there is currently no comprehensive overview of the literature available on migration health ethics in SEA. This background paper seeks to answer two key research questions:

1.   What is the existing literature on migration health ethics in SEA, and what are the key themes?

2.   What are the research gaps in migration health ethics?

To answer these questions, we conducted a scoping review based on the Joanna Briggs Institute Manual ( Peters *et al.,* 2020) and supplemented our understanding of the research gaps by engaging regional stakeholders in migration health. The findings would assist in establishing the future research agenda and priorities for scholars and researchers who are interested in research on migration health ethics in the context of SEA.

### Search strategy

We conducted a scoping review of the literature relating to three broad concepts: population (i.e. stateless population, migrants, refugees, asylum seekers, internally displaced people), issues (i.e. healthcare and ethics), as well as geographical contexts (i.e., 11 countries in SEA). The search strategy ( Table 2), inclusive of keywords and free text terms, was developed and subsequently used for the following electronic databases: PubMed, the Cumulative Index to Nursing and Allied Health Literature (CINAHL), and Web of Science. The search string was run for all fields. As no single database could comprehensively capture all bioethics research, the databases were chosen to capture ethical issues or concepts from varied sources and include research and perspectives from allied health professionals. All three databases were searched from 2000 until May 2023 over a period of four months (February 2023 to May 2023). As bioethics is a developing discipline in Southeast Asia, year 2000 and beyond is selected to reflect current bioethics discourse in the region. Additional searches for both full-text peer-reviewed articles, book chapters, commentaries, or other relevant policy documents and reports were done through forward and backward citation searches with Google Scholar, the WHO Regional Library (South-East Asia Regional Office), International Organization for Migration (IOM) repositories, and the United Nations High Commissioner for Refugees (UNHCR). Using keywords such as the names of SEA countries, ‘migrants’ and ‘refugees’, six bioethics journals (Journal of Medical Ethics, BMC Medical Ethics, Developing World Bioethics, Bioethics, Public Health Ethics and Asian Bioethics Review) were hand-searched for relevant publications. All searches were conducted in English.

**Table 2.** Search strategy.

**Search** ** strategy**	(asylum* OR migrant OR migration* OR refugee* OR displaced OR foreign* OR stateless OR non-citizen OR noncitizen OR mobile OR traffick*) AND (health*) AND (Ethic* OR Research ethics OR Biomedical ethics OR Bioethics)
**Inclusion** ** criteria**	1. Studies on the ethics of healthcare for or research with asylum seekers, refugees, internal and international labour migrants, internally displaced persons, mobile populations who are marginalised and/or vulnerable. 2. Studies conducted in SEA or from SEA perspective, including cross-national studies. 3. Includes primary and secondary research 4. Studies published in the English language from the year 2000-2023, to ensure relevance and accuracy in the interpretation of articles.
**Exclusion** ** criteria**	1. Study population involving international students, international expatriates, tourists, healthcare professional migrants. 2. Studies on SEA migrants resettled in countries outside of SEA. 3. Studies not based in SEA. 4. Epidemiological and socio-epidemiological studies 5. Did not contain substantial bioethical analysis

### Analysis

SHN independently reviewed the literature and identified relevant articles based on the title and abstract. ZLO and TCV assisted in assessing the articles’ full text to determine whether they fulfilled the inclusion criteria ( Table 2). Data was initially charted by SHN into a table with the following data items: first author, title and DOI, year, country, study area/population, study design, and remarks (brief summary and any key concepts), then were categorised into subdomains of ‘research ethics’, ‘clinical ethics’, ‘public health ethics’, and ‘general (unclassified)’.

The full-text articles were subsequently extracted and imported into NVivo 14 (Version Number: 14.23.0 (13)) to facilitate further thematic analysis using an iterative, inductive-deductive approach. Taguette ( https://www.taguette.org/), a freely accessible software, is also capable of the same analysis used in this study. Of note, the analysis modality was not decided at the outset because this is partly dependent on the types of bioethics research available, their study design, and any pertinent overarching theoretical/ conceptual framework should we come across it in this scoping review. We eventually decided on thematic analysis, due to the qualitative nature of articles retrieved, and the flexibility of thematic analysis in the absence of an overarching theoretical or conceptual framework. Starting with preliminary main codes, which comprised the ethics sub-domains, new codes were then added as sub-category codes and new main codes as the analysis progressed. Thematic analysis was conducted as described by Braun and Clarke, where themes were identified and reported using six phases: (1) becoming familiar with the data, (2) generating initial codes, (3) generating initial themes, (4) developing and reviewing themes, (5) defining themes, and (6) producing the report ( Braun & Clarke, 2006).

As the articles retrieved varied in terms of research designs and data outcomes, we adopted a modified narrative synthesis approach, adapted from Deliz *et al*., 2020. First, each included study was classified into one of the sub-themes derived from thematic analysis. Each study was then summarised in turn, comparing themes within and between studies. Any studies that were especially innovative or largely representative of the sub-theme were highlighted.

## Results

**Figure 1.**
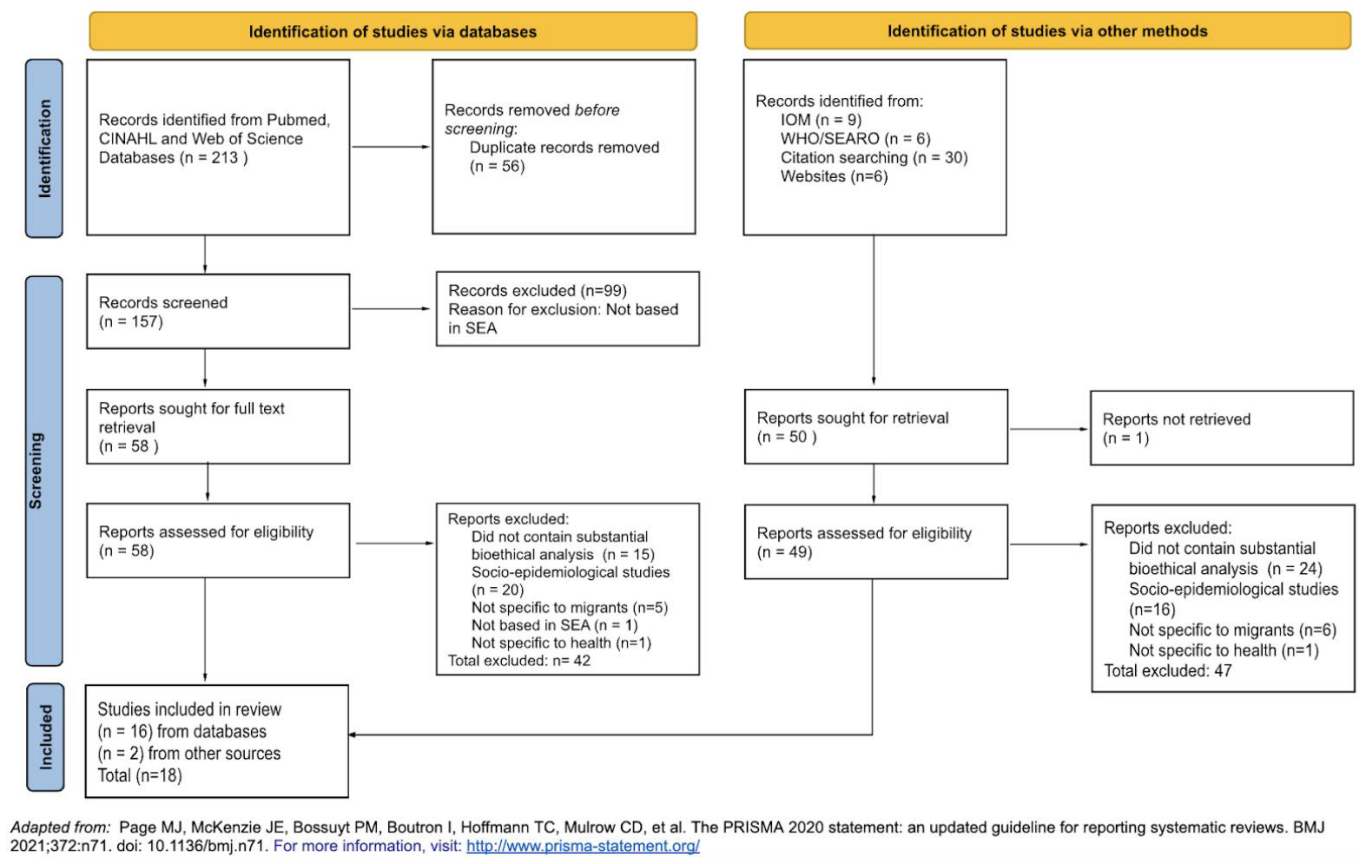
PRISMA Selection Flowchart.

212 potentially relevant journal articles were identified from three separate databases. Records were imported into Zotero ( https://www.zotero.org/) and deduplicated (n=157). These 157 articles were assessed against the exclusion criteria, and 99 articles were excluded as they were not based in SEA. A total of 58 full texts were retrieved and screened, and a further 42 articles were excluded (did not contain substantial bioethical analysis, n=15; socio-epidemiological studies, n=20; not specific to migrants, n=5; not based in SEA, n=1; not specific to health, n=1). Two additional publications were retrieved from citation search. A total of 18 studies were included in the final review (see Figure 1). More than 50% of the included studies were set in Thailand (n= 10), followed by Singapore (n=7) and Malaysia (n=1), with notable absence of research retrieved from predominantly migrant-sending countries such as the Philippines, Myanmar and Indonesia. There were 12 qualitative studies, 3 commentaries, and 3 case studies or narrative reviews. All research included pertains to adult international migrants. From the retrieved articles, only studies from Thailand adopted a participatory research approach, characterised by greater and more direct involvement of migrants in research processes, through the formation of a Community Advisory Board (CAB).

All publications included are summarised in Table 3.

**Table 3.** Included studies (in alphabetical order based on first author’s name).

	First Author	Title and DOI	Year	Country	Study Area/Population	Study Design	Remarks
1.	Adams, P.	Ethical issues in research involving minority populations: The process and outcomes of protocol review by the Ethics Committee of the Faculty of Tropical Medicine, Mahidol University https://doi.org/10.1186/1472-6939-14-33	2013	Thailand	Research with minority populations including migrants	Quantitative and qualitative analysis of submitted research proposals	Ethical issues in research with minority population
2.	Cheah, P. Y.	Community engagement on the Thai-Burmese border: Rationale, experience and lessons learnt. https://doi. org/10.1016/j.inhe.2010.02.001	2010	Thailand	T-CAB at SMRU	Descriptive study	Experience, lessons learnt and the unique challenges faced working with CAB
3.	Chin, C.	Precarious Work and its Complicit Network: Migrant Labour in Singapore https://doi.org/10.1080/00472336.2019.1572209	2019	Singapore	Migrant care workers and construction workers	Narrative review	Introduced the concept of precarity and complicity in migrant workers.
4.	Ditton, M. J.	The Control of Foreigners as Researchers in Thailand. https://doi.org/10.1525/jer.2009.4.3.49	2009	Thailand	Migrants at Thai- Myanmar border	Commentary	Challenges faced when conducting research with migrants.
5.	Dutta, M. J.	COVID-19, Authoritarian Neoliberalism, and Precarious Migrant Work in Singapore: Structural Violence and Communicative Inequality https://www.frontiersin.org/ articles/10.3389/fcomm.2020.00058	2020	Singapore	South Asian migrant workers in Singapore	Qualitative Research	Low-wage contract-based workers perform ‘hyper- precarious’ work, and how a lack of infrastructures for living and communication contributed to structural violence.
6.	Freeman, T	At the limits of “capability”: The sexual and reproductive health of women migrant workers in Malaysia https://doi. org/10.1111/1467-9566.13323	2021	Malaysia	Women migrant workers and stakeholders including health-care providers, unions, NGOs, employers and relevant government departments	Qualitative study	Conceptualisation of capability approach and application to migrant’s women access to sexual reproductive health.
7.	Jecker, N. S.	Justice and global care chains: Lessons from Singapore. https://doi.org/10.1111/dewb.12213	2019	Singapore	Migrant care workers	Qualitative study	Utilised the human capability approach to identify ways the capabilities were not upheld.
8.	Khirikoekkong, N.	Research ethics in context: Understanding the vulnerabilities, agency and resourcefulness of research participants living along the Thai–Myanmar border. https:// doi.org/10.1093/inthealth/ihaa052	2020	Thailand	Pregnant migrant women along Thai-Myanmar border	Qualitative study	Explored vulnerabilities and agency of clinical trial’s pregnant research participants
9.	Khirikoekkong, N	Culturally responsive research ethics: How the socio-ethical norms of Arr-nar/Kreng-jai inform research participation at the Thai-Myanmar border. PLOS Global Public Health, 3(5), e0001875. https://doi.org/10.1371/journal.pgph.0001875	2023	Thailand	community advisory board members, healthcare workers from the same clinic, or researchers st SMRU	Qualitative study	Identified how socio- ethical norms of Arr-nar/ Kreng-jai inform research participation at the Thai- Myanmar border
10.	Maung Lwin, K	Motivations and perceptions of community advisory boards in the ethics of medical research: The case of the Thai-Myanmar border https://doi.org/10.1186/1472-6939-15-12	2014	Thailand	T-CAB at SMRU	Qualitative study	Identified considerations relevant to the development of an approach to evaluating community engagement in complex research setting
11.	Parker, M. J	Moral and scientific boundaries: Research ethics on the Thai–Burma border. https://doi.org/10.1136/medethics- 2012-100582	2012	Thailand	Research with migrants at Thai-Myanmar border	Commentary	Overview of issues in research ethics.
12.	Pratt, B	Closing the translation gap for justice requirements in international research. https://doi.org/10.1136/medethics- 2011-100301	2012	Thailand	Karen and Myanmar refugees and migrants.	Case study qualitative research - in-depth interviews with trial stakeholders, direct observation at trial sites and analysis of trial- related documents	Empirical bioethics research to investigate if research at SMRU upholds obligations of justice in international research.
13.	Pratt, B	Linking international clinical research with stateless populations to justice in global health. https://doi.org/10.1186/1472-6939-15-49	2014				
14.	Pratt, B	Exploitation and community engagement: Can Community Advisory Boards successfully assume a role minimising exploitation in international research? https://doi. org/10.1111/dewb.12031	2015	Thailand	T-CAB at SMRU	Qualitative research - in- depth interview, direct observation, and document analysis	Used a case study approach to determine if members of T-CAB are able to safeguard communities from exploitation from research.
15.	Schaefer, G. O.	Zero COVID and health inequities: Lessons from Singapore. https://doi.org/10.1136/medethics-2022-108205	2022	Singapore	Migrant workers	Commentary	Public health strategy and ethics
16.	Tam, WJ	健康是本钱 - Health is my capital: a qualitative study of access to healthcare by Chinese migrants in Singapore. https://doi.org/10.1186/s12939-017-0567-1	2017	Singapore	Chinese migrant workers	Qualitative study	Descriptive study of migrants’ vulnerability through their migration journey
17.	Voo, T	Ethical medical repatriation of guest workers: Criteria and challenges. https://doi.org/10.1111/dewb.12286	2021	Singapore	Migrant worker with non- work-related injury	Case study	Normative analysis of ethics of medical repatriation
18.	Yea, S.	The produced injured: Locating workplace accidents amongst precarious migrant workmen in Singapore. https://doi.org/10.1016/j.socscimed.2022.114948	2022	Singapore	Migrant workmen men who were out-of-work due to an injury or salary/labour problems with their employer in Singapore	Qualitative study	High injury rates are caused by migrants' structural and economic vulnerabilities.

We found a number of researchers who have conducted their empirical research or analysis through the lens of these ethical concepts: (i) capability, agency, and dignity (n=2); (ii) vulnerability (n=4); and (iii) precarity, complicity, and structural violence (n=3).

We also found research articles and commentaries which did not rely on specific ethical concepts in their methodological approach or analysis. These articles correspond to subfields in bioethics, namely: (i) research ethics (n=9) which concerns issues in the conduct of research; (ii) clinical ethics (n=1) which concerns issues in healthcare; and (iii) public health ethics (n=1) which pertains to ethical issues in public health ( National Institute of Environmental Health Sciences, n.d.).

Although not specifically analysed in this review, there were significant numbers of publications which focused on health equity or were motivated by inequity in migrant health (n=26) (see Shu, 2023a). These papers typically revealed inequities in health status or determinants between migrant and host populations.

### Ethical concepts

The ethical concepts discussed in this section, while presented as distinct entities for the purpose of clarity, are in fact interlinked with many shared features.

***Capabilities, agency, and dignity***

We found two studies which analysed and applied the ethical concepts of capabilities, agency, and dignity in their research.

Freeman *et al.* (2021) applied a capability approach (CA) as a conceptual framework to analyse the experience of women migrant workers in Malaysia in meeting sexual and reproductive health (SRH) needs. The CA focuses on expanding the freedoms and opportunities (“capabilities”) available to individuals to live a life they value, including in the pursuance of SRH. The authors also considered “agency freedom” (i.e., “freedom to bring about the achievements one values and which one attempts to produce”) and “agency achievement” (i.e., the realisation of these pursuits) as a requisite to human flourishing ( Sen, 1992, pp.56–57). Using a qualitative approach, the authors found that several capabilities that are critical in the management of SRH include having opportunities to acquire SRH knowledge, freedom to access SRH health care, and the presence of community leaders to act as focal points to share and disseminate SRH information. Through this approach, barriers to accessing SRH were identified, and essential resources were identified to enhance SRH amongst women migrant workers.

Similarly, Jecker and Chin (2019) also employed a human capability approach in analysing the plight of domestic care workers from Indonesia and the Philippines in Singapore. The authors flesh out the concept of ‘human dignity’ as respect owed to other persons simply by their virtue of being human, and that is independent of our favourable appraisal of a person's merits. Thus, ‘human dignity’ is linked to what being human means (i.e., what humans are capable of doing and being), and it includes being able to “have health, nourishment and shelter” and “to use one’s body to do what one intends to do” ( Jecker & Chin, 2019, p.159). The study found that human dignity comes under threat for domestic care migrant workers in Singapore as they lack minimal capability for health and bodily integrity. Movement restrictions and mandatory biannual health checks, which closely relates to privacy, autonomy and agency, was not respected. ^4^ A multifaceted failure to safeguard the dignity of domestic care migrant workers was highlighted and recommendations to protect their capabilities and dignity were proposed.

***Vulnerability***

There are four papers which examine ethical issues involving migrants in different contexts using the concept of vulnerability.

In their qualitative study with pregnant migrant women living along the Thai-Myanmar border, Khirikoekkong *et al.* (2020) uses the definition of vulnerability as a state of being “more susceptible to risks and less able to protect one’s own interests”, where “being in a vulnerable situation shapes obligations for others to help or take special care”, to consider ethical issues with research with this migrant population. The authors suggest that labelling a study population as ‘vulnerable’ (such as ‘pregnant women’, ‘migrants’ or ‘children’) can potentially err in two ways: first, by unfairly excluding individuals who are capable of consenting or assenting (with some support) and depriving them of the potential benefits of research participation, and secondly, by not being protective enough of individuals who do not belong to designated vulnerable groups. The study identified political, economic, social and health vulnerabilities, and found that despite the challenges and vulnerabilities in everyday lives, migrant women were able to exercise resourcefulness and agency to benefit from research participation. Therefore, researchers should be aware of specific contextual and structural vulnerabilities and be responsive to the vulnerabilities by respecting the agency of migrant women, minimising the burden of research participation, and providing adequate compensation.

Although the term ‘vulnerability’ was not specifically defined, Freeman *et al.* (2021) described women as “more vulnerable [compared] to their male counterparts” because “only women experience pregnancy and childbirth, sexually transmitted infections (STIs)…and women are more likely to be subjected to sexual violence’, and ‘these vulnerabilities are magnified in the context of migration”. Moreover, women migrant workers who are pregnant or have contracted STIs are liable to work permit cancellation and those who stay on become undocumented. Consequently, pregnant migrant workers may risk their lives inducing abortions for fear of losing their jobs. The risk of deportation following migrant care workers’ pregnancy was also highlighted by Jecker and Chin’s paper which emphasises gender-based vulnerabilities ( Jecker & Chin, 2019). Similarly, Tam *et al.,*’s research on Chinese migrant workers in Singapore highlighted different points of vulnerability through their migration journey, from existing vulnerabilities during the migration process, through their point of injury or illness and healthcare consultation, to the point of discharge, recovery and repatriation ( Tam *et al.,* 2017).

***Precarity, complicity and structural violence***

Expanding on the concept of vulnerability, three other studies analysed the concept of precarity, complicity and structural violence with respect to the plight of migrant workers in Singapore. 

Chin introduced the concept of precarity and complicity in the management of temporary migrant labour in Singapore ( Chin, 2019). Precarity is defined as a set of vulnerable labour conditions, including “low wages, short fixed-term contracts, numerous intermediaries such as recruitment agencies and sub-contractors, and poor legal and social protections”. Vulnerability in this context is defined as ‘a state of being exposed to the possibility of harm’ by exploitation, abuse and injury. With a focus on male construction workers and female domestic workers in Singapore, Chin provided a framework to identify how migrant workers' management strategies increase social vulnerabilities that constitute precarious work. Moreover, migrant workers were dependent on employers for food and accommodation, effectively deprived of ‘ordinary means’ to fulfil their well-being. The author assigned moral responsibility to the complicit network, i.e., the state, employers, agents and others who enable, collaborate and condone social vulnerabilities, and identified grounds for redress.

Yea provided a conceptual framework for the ‘produced injured’ – emphasizing that ‘the political economy of migrant labour increases vulnerability to injury’ and fatalities among migrant workers ( Yea, 2022). Using qualitative research with South Asian migrant workmen, Yea highlighted that the organisation of migration (including debts and deportability) and deceptive recruitment practices (including wrongful deployment and substandard living conditions) have contributed to increased risks for injury. Dutta similarly described how low-wage contract-based workers in Singapore perform ‘hyper-precarious’ work – defined by a lack of protection strategies, an absence of systemic infrastructures for workers to address their labour-related needs, and policy oversight that holds the employers accountable ( Dutta, 2020). Structural contexts of poor housing, sanitation infrastructures and food insecurity are framed as a form of ‘structural violence’ that worsens migrant workers' health and well-being, particularly during the COVID-19 pandemic.

### Research ethics

Nine articles pertaining to ethical issues with migrant health research in Thailand were included in this review. This section could be broken down into the sub-themes of (i) general commentary (n=2), (ii) research ethics committee (n=1), (iii) community engagement (n=3), (iv) cultural responsiveness in research (n=1), and (v) research and global health justice (n=2).

***General commentary***

Drawing on the experience of malaria research at the Thai-Myanmar border, Parker provided a snippet of the complex cluster of challenges in research ethics ( Parker, 2012). Researchers are often faced with conflicting forms of guidance, with challenges in establishing good practices and effective solutions in research, taking into account religious, political, linguistic, and ethnic diversity. There can also be concerns about the scope of the responsibilities of researchers before, during and after research, alongside difficulties in navigating the competing interests of research stakeholders such as government agencies and research funders.

Ditton and Lehane highlighted the need to understand the complexity of the political environment, cultural nuances and the roles of various stakeholders in chronic humanitarian crises involving migrants ( Ditton & Lehane, 2009). The authors have also documented the process of obtaining research approval, as well as ethical and legal considerations when working with all stakeholders along the Thai-Myanmar border.

***Research ethics committee***

Adams *et al.*’s (2013) paper provides an overview of the common research ethics issues encountered by a research ethics committee. Specifically, it analysed research proposals involving minority populations (including migrants) submitted to the institutional ethics committee of the Faculty of Tropical Medicine, Mahidol University, Thailand. The research found that the majority of proposals submitted required revision or deferrals to ensure that there is no exploitation and undue negative legal implications. The authors suggested the establishment of a community advisory board consisting of relevant community members in the early planning phase to ensure that research activities achieve acceptable ethical standards.

***Community engagement***

In safeguarding migrant communities’ interests, the Shoklo Malaria Research Unit (SMRU) at the Thai-Myanmar border facilitated the set-up of the Tak Province Community Ethics Advisory Board (T-CAB) in 2009 ( Cheah *et al.,* 2010). Extensive training was provided to committee members consisting of Burmese or Thai nationals from the Karen ethnic group to undertake the tasks of vetting through research proposals. The T-CAB serves as an independent research advisory committee and acts as a bridge between researchers and community members, ensuring that local cultural sensitivities are taken into consideration. Despite the challenges in setting up a community advisory board, there were significant benefits through engagement with a wide range of community members. Besides advising on the ethical and operational aspects of research, the advisory board also serves as a platform for multilateral communication between researchers, research participants and other community members, and has an influence on research aims and impact. Further research has been done to investigate whether such an advisory board is reciprocally beneficial for the T-CAB members ( Maung Lwin *et al.,* 2014). Despite practical challenges in navigating communication amongst board members of different cultural and linguistic backgrounds, evidence suggests that the T-CAB members saw positives in being active participants of research as they were able to serve as gatekeepers and empower the community by ensuring that research responds to the communities’ genuine needs ( Maung Lwin *et al.,* 2014).

Two years after the establishment of the T-CAB, Pratt *et al.* investigated if the members could effectively safeguard research communities against exploitation ( Pratt *et al.,* 2015). It has been proposed that the role of reducing exploitation should be formalised, and training needs to be provided for the members to develop critical appraisal skills and an understanding of what constitutes ‘exploitation’ and ‘health priority’. At the time of the study, the T-CAB members did not feel that they had the authority to take action if their recommendations were not adhered to. In recognition of the inherent power imbalances and resource limitations in such research settings, the study found that a considerable amount of time and training is needed to develop core competencies and capacities in protecting research communities against potential exploitation.

***Cultural responsiveness in research***

In recognition of the lack of practical research ethics guidance which takes into account moral perspectives in diverse cultural contexts, an empirical sociocultural analysis was performed using a qualitative approach at the Thai-Myanmar border ( Khirikoekkong *et al.,* 2023). The research specifically analysed how sociocultural-moral norms pervasive in all aspects of the lives of local communities, known as *Arr-nar* (in Burmese and Karen) or *Kreng-jai* (in Thai), influence multiple aspects of research ethics, including “voluntary participation, provision of fair benefits, and understanding of research risks and burdens”. The study detailed the inherent complexities in the direct translation of ethical concepts into local languages, and the various steps taken to ensure the accuracy of the interpretation of the research data. The study also incorporated a creative use of participatory visual methods using drawings and stories to confirm themes emerged from the qualitative data.

The complex socio-ethical norms of *Arr-nar/Kreng-jai* not only inform ethical responsibilities, such as ensuring understanding, voluntariness, or duty of care, it also informs moral feelings, such as trust, appreciation, guilt or concern. *Arr-nar/Kreng-jai* impacted the motivations behind research participation, the commitment to maintaining research participation, and was implicated in the assessment of voluntariness, understanding and refusals. The research indicated that a greater sensitivity to local moral and social norms can be central to ensuring the ethical conduct of international research, and offered practical ethical guidance for researchers working in SEA.

***Research and global health justice***

‘Justice’, in the context of research ethics, often refers to the fair selection and recruitment of research participants, as well as equitable distribution of burdens and benefits ( Benatar, 2002). In global health research, this means ensuring that the research burden and risks should not fall disproportionately on participants in resource-poor context, and correspondingly research benefits should not be conferred disproportionately on those residing in the ‘global north’ where research funders and research institutions concentrate. The ethical framework of ‘research for health justice’ describes how international clinical research should be organized to advance the ends of global justice ( Pratt *et al.,* 2014). Pratt *et al.,* detailed the main requirements of achieving justice in international clinical trials, and laid out the obligations of governments, funders, sponsors, researchers and global health institutions. Clinical research in low and middle-income research settings commonly includes an obligation to:

i) respond to local health needs and priorities
ii) ensuring the provision of ancillary healthcare
iii) provide access to post-trial benefits such as treatment or practices developed by the research
iv) strengthen research capacity in host countries and communities.

Using a case study approach at SMRU, in-depth interviews (with study investigators, T-CAB members, trial participants and research funding representatives), direct observation of T-CAB meetings and trial sites, and analysis of trial-related documents were conducted to assess whether and how obligations of justice are translated into international research practice ( Pratt *et al.,* 2012). The study found that external research actors from high-income countries were partially able to uphold global health justice obligations. Challenges included the difficulties for external researchers to keep up with the changing disease burdens of host communities and adjust the focus of their research agendas accordingly. Moreover, sustainable research capacity building amongst mobile populations was not feasible due to regular staff turnover as a result of resettlement or migration. The study also highlighted difficulties in the provision of post-trial benefits when the population is not able to access state health systems.

***Clinical ethics***

Voo *et al.,* performed a normative analysis of ethical issues concerning the medical repatriation of documented migrant workers in Singapore who sustained non-work-related health conditions ( Voo *et al.,* 2021). The legal and policy regime in Singapore creates a landscape where migrant workers who suffer serious non-work-related medical conditions are vulnerable to repatriation. By adapting Kuczewski's ethical criteria for medical repatriation of undocumented migrants in the US ( Kuczewski, 2012) and applying them to a case study, critical aspects of the professional and ethical duties of medical professionals were discussed. In a context where migrant healthcare is largely dependent on ‘employer’s responsibility’, medical professionals should nonetheless avoid acquiescing to an employer’s decision which is against the best interests of the migrant worker qua patient. The authors also listed a set of assessment questions to assist medical professionals in adhering to ethical standards in medical repatriation to avoid ‘patient-dumping’ in migrant-sending countries.

***Public health ethics***

Schaefer commented that the zero COVID policy as a public health strategy in Singapore exacerbated existing health inequalities for migrant workers in Singapore ( Schaefer, 2022). Restrictive policies curtailed their ability to earn a living and forced them to remain in cramped living spaces for extended periods of time. Despite being vaccinated, migrant workers’ movement remained limited even as restrictions were eased for the general population, raising doubt if public health strategy is truly prioritizing and promoting health equity within its broad framework.

## Discussion

Whilst the health of migrants has been studied to varying degrees in SEA, our research on the ethical aspects of migration health highlights a gap in research capacity and output, especially in empirical bioethics research and normative analysis. In the process of conducting this review, we found a significant number of epidemiological and socio-epidemiological studies that seek to identify differences in health and health care for different groups of migrants ( Kim *et al.,* 2023; Pham *et al.,* 2019; Rajaraman *et al.,* 2020). A key motivation for doing epidemiological studies, which often seeks to identify disparities in different groups, is often an ethical one with aims to bring about greater health equity between host and migrant communities ( Wild & Dawson, 2018). Socio-epidemiological studies are also useful in examining the interaction between socio-structural factors, distribution of health and disease, as well as how these influence individual and population health ( von dem Knesebeck, 2015). Nonetheless, we noticed an underapplication of ethical theories or frameworks, or conceptual analysis.

### Utility of bioethics research

By utilising conceptual tools, ethical issues experienced in practice by the stakeholders involved can be better articulated, and consequently, could more reliably encourage and support responsible ethical conduct ( Beauchemin *et al.,* 2022). Ethical concepts such as agency, vulnerability, precarity, and complicity identified from this review allow stakeholders in migrant health to identify and understand complex ethical issues in migrant health policy and research. For instance, the research by Chin and Yea has shown that the conceptualisation of the ethical issues faced by migrants may provide grounds for assigning responsibilities, reform and redress ( Chin, 2019; Yea, 2022). The research by Jecker and Chin (2019) and Freeman *et al.* (2021) similarly provided evidence of ways in which human capabilities were not upheld for migrant women in Singapore and Malaysia respectively. This knowledge may then be used for further analysis of policies and development of practices that promote well-being and dignity ( Molenaar & Van Praag, 2022).

Using the concept of vulnerability as an example, migrants are considered to be vulnerable due to poverty or low socioeconomic status, precarity, poor access to healthcare, and discrimination. The use of such terms can be useful in describing the practical difficulties migrants face, but the widespread generalisation in the use of ‘vulnerability’ as a fixed descriptor for migrants without clear understanding of how they are or are *not* vulnerable may have potentially negative implications ( Molenaar & Van Praag, 2022). This approach often fails to consider the perspectives and experiences of the migrants themselves, resulting in their marginalisation or exclusion from discussions on their health and health access. The presumed inherent vulnerability could also disproportionately redirect focus on migrants’ perceived deficit and weakness, and draw attention away from the structural causes of vulnerability which could be remedied ( Molenaar & Van Praag, 2022). It may even affect migrants themselves – some may refrain from engaging in positive, constructive activities, consciously limiting their abilities in a bid to portray themselves as vulnerable ( Mohammadi & Askary, 2022). In research, it could unfairly exclude their participation or could undermine their agency, denying migrants the opportunity to be involved in beneficial health research ( Khirikoekkong *et al.,* 2020).

The theorisation of migration health ethics may also help us to understand the reasonings behind actions (or inactions) and could form the basis of why we should care enough about migrant health and act on it. Empirical bioethics research with an aim to describe policies and practices would assist in the identification of ethical issues and how they are experienced in practice. As evidenced by the publications included in this review, empirical research allows for an insight into how ethics is being carried out and whether or not a research or an intervention is upholding ethical standards ( Cheah *et al.,* 2010; Pratt *et al.,* 2014). Pratt *et al.,* have provided evidence that bioethics empirical work is necessary to inform the obligations of justice in international clinical research and in the development of guidance to facilitate adherence and implementation of ethical guidelines ( Pratt *et al.,* 2014). These play a crucial role in promoting justice in global health because only by having empirical evidence of the real and potential problems of putting ethics into practice can we then seek to address the issues identified and work towards improvement. Research detailing the actual experience of the implementation of a CAB, its challenges and benefits including from the perspective of community members themselves can also benefit other researchers in the region seeking to implement CAB to ethically enhance the way in which they conduct research ( Cheah *et al.,* 2010).

Countries in SEA may share geographical proximity, but each country is rich with its own ethnic, religious, linguistic and cultural traditions and norms ( Arphattananon, 2021). Even within the same national territory, there may be a divergence in socio-cultural norms and values. As evidenced by the research on cultural responsiveness by Khirikoekkong *et al.* (2023) empirical ethics research on sociocultural and ethical norms can foster a nuanced comprehension of local customs and practices, and how these cultural factors impact the realm of research ethics. Knowledge from research as such may then aid in the development of research ethics support that is more responsive to local and regional ethical norms.

Normative ethics analysis, such as the study by Voo *et al.* (2021) allows for discussion of ethical concepts, their implications, and identification of what ‘should be’ done in potentially ethically challenging circumstances in migrant’s medical management. Based on other research done in SEA, medical professionals are often faced with ethical dilemmas such as being forced to make clinical decisions based on migrants’ ability to pay ( Loganathan *et al.,* 2019), having to navigate a dissonance between laws and professional codes including but not limited to the obligation to report undocumented migrants to the police ( Chuah *et al.,* 2019) and being compelled to restrict the issuance of medical certificates ( Loganathan *et al.,* 2019; Voo *et al.,* 2021). Ethical guidance in migrant health delivery could be useful in assisting medical professionals in confronting multifaceted ethical dilemmas.

The health and well-being of migrants are impacted by a complex range of global and local factors, both within and beyond the health sector. Therefore, an ethical approach to policy making, research and clinical practice can provide means to identify ethical issues, frameworks for systematising information and catalyse the formulation of ethically acceptable solutions ( Onarheim *et al.,* 2021). The systematic unpacking of dilemmas, values, and conflicts of interest equips decision-makers in migration health with analytic tools and guidance in dealing with challenges in policy making and practice. Ethical scrutiny and procedural guidance can also assist in highlighting policies and practices that are at variance with fundamental principles of medical ethics, public health and human rights ( Onarheim *et al.,* 2021).

### Other research gaps

A bibliographic analysis of global migration health research from 2000 to 2016 has shown that most of the published academic literature represents the perspectives of high-income migrant destination countries, with a focus on communicable health diseases and mental health ( Sweileh *et al.,* 2018). Despite the fact that Asia has one of the largest numbers of international migrants, research output from Asia is relatively low in comparison to other parts of the world ( Sweileh *et al.,* 2018). Based on the limited number of studies identified and included in this review, there is a need to increase funding, human resources and research output in migration health ethics in this region. We found that the majority of bioethics research included in this review was done in Thailand, Singapore and Malaysia, which are major migrant destination countries in SEA. There is a paucity of migration health ethics research found in other parts of SEA, which is indicative of the need for future research and collaborative work.

We engaged with regional stakeholders in migration health to supplement our understanding of the research gaps identified from this review, which are detailed below:

***i)   Research involving migrant children***

As most migration health research was undertaken in high income countries, issues relating to migrant children in developing countries remain largely unexplored ( Sweileh *et al.,* 2018). This review has similarly identified a dearth of research in ethics relating to migrant children. There are millions of child migrants who travel across borders with or without their parents or were born to migrant parents in host countries ( ILO, 2015). Children who are unaccompanied or undocumented can be at increased risk of exploitation with limited access to basic social services such as education, housing and healthcare ( ILO, 2015). Considering the multifaceted realities of migrant children, more research could also be done to contextualise the practical difficulties in conducting health research and providing health care for migrant children in SEA. Such documentation is crucial in the formulation of responsive and pragmatic ethical guidelines to support future research that seeks to advance migrant children's health and well-being.

***ii)   Research from migrant-sending countries***

The discourse about migration health ethics has focused on issues related to barriers to accessing health care, with a handful of studies focused on individual migrants’ well-being and dignity in destination countries. In predominantly migrant-sending countries such as Indonesia, Cambodia, and the Philippines, more research could be done to document migrants' experiences or challenges in destination countries or on return to their home countries. 

***iii)   Quality of migrant healthcare***

In investigating the ethical aspects of health service delivery, further research could focus on the quality of health care – from the perspective of service providers and migrants. Such studies can be useful for quality improvement and could serve as a baseline for subsequent monitoring and evaluation initiatives. Analysis of culturally competent, migrant-inclusive health systems is crucial so that different policy options can be considered to realise “migrant-inclusive universal health coverage” systems regionally ( Pocock *et al.,* 2020). Studies on policy implementation, such as research from Thailand ( Suphanchaimat *et al.,* 2019) which investigates the divergence between intention and implementation of health policies for migrants may be useful in the improvement of health services for migrants.

***iv)   Participatory health research***

Participatory health research with an aim to shift the research paradigm from “research on people” to “research with people” is gaining international recognition and traction ( WHO, 2022a). One expression of participation is community engagement across the different phases of research, especially in advising on research ethics, and not just tokenistic involvement of migrants in health research, where migrants’ participation is limited to initial consultations to support recruitment or for dissemination of information at the end of research cycles ( WHO, 2022b). The CAB in Tak Province, Thailand, is a notable example of good practice that promotes the participation of migrant community members in research advisory boards, advocating for a shift away from solely researcher-directed projects and outcomes ( Cheah *et al.,* 2010). There could also be a greater imperative to involve community members not just as ancillary support (for example, in participant recruitment and interpretation services) but to formally embed their roles in the ethics committee and be involved as researchers themselves.

***v)   Research with internal migrants***

The vast majority of individuals do not move across national borders; instead, a significantly higher number migrate within their own countries. In 2009, approximately 740 million people were internal migrants, which is over three times the number of international migrants. ( McAuliffe & Triandafyllidou, 2021). However, most studies have focused on the ethics of the health of international migrants, indicative of a potential need to also focus on the ethical challenges stakeholders face in the health and healthcare for internal migrants.

### Strengths and limitations

This scoping review – the first of its kind for SEA – aims to map out and signpost existing bioethics research in migration health for the region. Given the paucity of bioethics research in this area, the findings of this review are ameliorated and complemented by engagement with key regional stakeholders and experts in migration health in SEA. As bioethics is a relatively new field in SEA, this review showcases the range and utility of bioethics research on migration health, and demonstrates some ways in which it could contribute to the advancement of migration health regionally.

However, this study possesses several limitations. Firstly, in terms of search strategy, the selected search terms were broad and might not have captured all relevant keywords in bioethics, leading to the potential exclusion of important bioethics research in SEA. The broad search terms may have also led to the limited size and composition of the review’s outcome.

Due to limitations in language interpretation, non-English publications were not included in this review. Secondly, this review does not differentiate between distinct migrant groups in different countries. Migrants are not a homogeneous group, and thus specific contextual factors, such as geography, socio-politics, and legal considerations, should be considered when examining their health outcomes, structural vulnerabilities and ethical issues in research and health practices involving migrants. Future work needs to be done to identify and analyse a broader body of research, and to identify further research gaps or priorities.

## Conclusion

More robust research is required to understand the multidimensional ethical aspects of migration health in SEA. Based on our review and engagement with regional experts, we conclude that bioethics scholarships have an undeniably crucial role in synthesizing the content and extent of the ethical obligations of different stakeholders in migration health and in the construction of context-specific ethical guidelines or frameworks to safeguard and support all stakeholders in migration health. As Onarhaim *et al.* proposed, ethics research and normative work could assist decision-makers, be it in health policy, healthcare or research, by developing “methods to identify ethical issues, frameworks for systematising information and suggesting ethically acceptable solutions, and guidance on procedural concerns and legitimate decision-making processes (Onarhaim *et al.*, 2021). Our review found growing work in these areas in SEA, albeit concentrated in particular countries, such as frameworks to identify and mitigate the vulnerabilities of migrant workers. Clarity in ethical concepts is critical for migrant health by providing insights into the moral dimensions of the issues and challenges and is a cornerstone in the development of ethical frameworks for practice. In generating ethical solutions to issues relating to migration health in the SEA context, it is crucial for new knowledge to be generated through research grounded in migrants’ realities, with emphasis given to regional and temporal contexts ( WHO, 2022a).

The convergence of migration policy with ethics and health created a growing imperative for policy-makers, scholars, and practitioners in SEA to engage in cross-sector dialogue to align priorities and address the complexities of migrants’ health ( Kapilashrami *et al.,* 2020). It is of paramount importance that ethics, in consideration of relevant regional values and belief systems, is central to research, policies, interventions, healthcare delivery and community engagement systems. As such, it would require regional expertise and meaningful collaboration to develop research agendas that ultimately aim to improve long-term health outcomes for migrants. The Southeast Asia Bioethics Network aims to cultivate the development of a vital interdisciplinary research community with a diverse range of skills and expertise, and positions itself as a platform to engage and connect various stakeholders in migration health ethics and research. Ultimately, it aims to advance the health of migrants in the region through the promotion of ethical considerations in policy development, research, and health practices.

## Data Availability

Zenodo: Publications with focus on health equity. https://doi.org/10.5281/zenodo.8202416. ( Shu, 2023a). Zenodo: Search Strings. https://doi.org/10.5281/zenodo.10071196. ( Shu, 2023c) This project contains the following underlying data: Appendix 1 Publications which focus on health equity, or were motivated by inequity in migrant health but were not included in analysis.docx. Zenodo: PRISMA-ScR checklist and flow chart for ‘Migration Health Ethics in Southeast Asia: A Scoping Review. https://doi.org/10.5281/zenodo.8226767. ( Shu, 2023b). Data are available under the terms of the Creative Commons Attribution 4.0 International license (CC-BY 4.0).
