# Supplementary material for: Migration health ethics in Southeast Asia: a scoping review
Source: Wellcome Open Res. 2024 Jun 4;8:391. Originally published 2023 Sep 1. [Version 3] doi: 10.12688/wellcomeopenres.19572.3 (PMC11002524; doi:10.12688/wellcomeopenres.19572.3)
Supplement: Peer review history

# Reviewer report: Reviewer response for version 3

**DOI:** 10.21956/wellcomeopenres.24721.r85728

**Published:** 2024-07-17

**Authors:** Ornella Punzo

**Affiliations:** 1 Istituto Superiore di Sanità, Roma, Italy

First of all, thank you for inviting me to review this paper and sorry for the delay in revising and approving the latest versions. 

 I praise the authors' efforts to revise the paper according to the 3 reviewers' remarks and reckon that the latest version is much improved compared to the original one. Therefore I am happy to approve this latest version.

Are the rationale for, and objectives of, the Systematic Review clearly stated?

Yes

Is the statistical analysis and its interpretation appropriate?

Not applicable

Are sufficient details of the methods and analysis provided to allow replication by others?

Yes

Are the conclusions drawn adequately supported by the results presented in the review?

Yes

Reviewer Expertise:

Migration and Health, Infectious Diseases Epidemiology, Climate change and Health

I confirm that I have read this submission and believe that I have an appropriate level of expertise to confirm that it is of an acceptable scientific standard.

# Reviewer report: Reviewer response for version 2

**DOI:** 10.21956/wellcomeopenres.22412.r76341

**Published:** 2024-04-08

**Authors:** David Ingleby

**Affiliations:** 1 University of Amsterdam, Amsterdam, Netherlands Antilles

I have been invited to peer-review this paper following reviews by two other experts and a revision of the paper in response to these reviews. To familiarise myself with the discussion so far I have read the other reviews and examined the changes between Versions 1 and 2 to see how the authors have responded to the reviews.

 Both reviewers had criticisms of the procedure used for retrieving articles, in particular the databases searched and the search terms employed. However, to do the searches differently the researchers would have to start again from scratch, which is probably impossible. The most that can be done with the reviewers’ methodological criticisms is to provide more justification or expand the section on limitations (as Reviewer 2 indeed recommends).

  

1. **Have the authors responded adequately to the reservations stated by the first two reviewers?**

**Introduction**

 Reviewer 2 suggested putting a footnote on internal migrants into the text, which has now been done. However, the text is still a bit confusing. Adding up the totals of international and internal migrants does not produce a revised estimate: it simply defines migrants differently. The IOM definition (“whether within a country or across an international border”) is at loggerheads with that used by UN DESA, which only counts international migrants. The authors should make clear that they use the IOM variant.

**Migration as a complex determinant of health**

 Reviewer 2 commented that this could be “reduced, better organized and made crisper, with clearer signposting”. The section has indeed been reduced by the deletion of four paragraphs, but nothing else has been changed. The first sentence, WHO’s well-known but controversial 1948 definition of health, could safely be removed. Table 1 (originally 2) gives an authoritative overview of the determinants of health in different phases of migration and should be kept. The section concludes with a paragraph about the role of health in the 2030 Agenda for Sustainable Development.

 Unfortunately, all that remains of this section are accurate but highly general statements showing that migration is indeed a complex determinant of health. All the material specific to SEA was in the four paragraphs that have been removed. This is unfortunate, because much of it would prepare the reader for the kinds of ethical issues that arise in the region. For example, the fact that only the Philippines, Cambodia, and Timor-Leste have ratified the 1951 Refugee Convention or its 1967 Protocol is a very important ingredient of the SEA context. The same applies to ‘families left behind’ and trafficking for labour and sexual exploitation.

 My suggestion is that rather than simply restoring the deleted material, the authors could weave it into  the previous section (Migration: A Southeast Asian context), where it might even be more appropriate.

**The role of bioethics in SEA migration health**

 Reviewer 2 called for explicit attention to “ethics around access to health, as well as research”, and two paragraphs about these themes have been added. However, as I note below (p. 1, Background), the health theme is broader than just access to healthcare – the reviewer referred to ‘access to health’, not to healthcare.

**Method**

 Reviewer 1 questioned the choice  of CINAL as one of the three databases selected. Reviewer 2 suggested that SCOPUS would be a better choice of database than CINAL Alternatively, this reviewer suggested that the “rationale behind choosing the final databases may be provided to strengthen the authors’ case.”

 Regarding the search terms, Reviewer 2 felt they were ‘limiting’, and gave examples of other terms that might be used in the literature to cover the concepts of interest. This is indeed normal procedure in a bibliometric analysis: if you assume that all authors use words in exactly the same way as you do, you may overlook discussions which deal with the topics you are looking for but use different terminology. However, realising that a new search might not be possible, Reviewer 2 wrote: “alternatively, elaborate the limitations of the search string and selected keywords, as well as the rationale behind the same.”

 In the revised version of this section, little attempt was in fact made to justify the choices made. An argument was given for consulting CINAL rather than only PubMed or Web of Science, but not for the limited search terms employed. If the bibliometric search had been the only method used to locate relevant articles, I feel that the risk of missing relevant articles would have been unacceptably high: but we are told that many other sources were consulted, as well as six bioethics journals. Perhaps for this reason, the response of Reviewer 2 to the revised version was conciliatory. This reviewer also expressed a wish for more clarity about the analysis procedure (were themes extracted, and if so, how), and suggested examining the ethical content of WHO's migration and health global research agenda.

 In my view, relevant discussions in the literature may have been missed because proponents of an ethical approach sometimes fail to recognise such an approach when they see it – simply because it may not be labelled as such. Concepts such as fairness and justice are often applied within a human-rights or public-health framework: this means that attempts to construct watertight boundaries around bioethics are doomed to failure. These frameworks are not mutually exclusive.

 It seems that only during the course of the research did the authors realise that an approach in terms of ‘health equity’ overlaps with bioethics. At the outset, ‘epidemiological or social-epidemiological’ approaches were systematically excluded from the study: to their credit, the authors seem to have realised that this was unjustified, so that in addition to the list of 18 articles chosen using the search procedure, another 26 articles were listed in Appendix 1 as “Publications which focus on health equity, or were motivated by inequity in migrant health but were not included in analysis”. I fail to understand why ‘equity’ was not admitted to the canon of ‘ethical concepts’, and why the 26 articles have been left unanalysed.

**Results**

 Reviewer 2 requested some content analysis at the beginning: this was provided. The question about the thematic/conceptual analysis was answered. Furthermore, the reviewer found the results sections a bit too long and monotonous in places. Accordingly, some (minor) cuts were made.

**Discussion**

 Reviewer 1 “sometimes found the connections between ethics and social determinants of health, including migrant health, a bit vague.”  In their reply the authors agreed with this characterisation, but found that the paper contributes to a solution by promoting research and analysis that clarifies these links.

 Reviewer 2 had requested that the authors should elaborate the limitations of the search string and selected keywords.  In the section on Limitations the authors concede that the search terms may have led to important research being missed, but they ascribe this to the terms being ‘too broad’. I think the reviewer meant the opposite: the terms were too narrow, because they failed to include synonyms or words or phrases similar to the concepts being searched for. 

 The second sentence of the Discussion runs: “In the process of conducting this review, we found a significant number of epidemiological and socio-epidemiological studies that seek to identify differences in health and health care for different groups of migrants (Kim et al., 2023; Pham et al., 2019; Rajaraman et al., 2020).” I assumed that this passage refers to the list of publications on health equity in Appendix 1 – but the articles by Kim et al. and Pham et al. are not to be found there. It's not clear what, if any, is the relation between the two lists.

**2. My own comments on version 2 of the article**

 Points that have already been made by the first two reviewers will not be repeated here. Page numbers in what follows refer to the pdf version.

 p. 1: Background. This section mentions only healthcare and research – public health has been left out. All too many publications discuss ‘migrants and healthcare’, overlooking the fact that migrants also need to be included in health promotion and education, screening programmes, population research and health policy in general. In this article ‘healthcare’ is mentioned 37 times: the authors need to check that the other issues are not being neglected. Talking about health services rather than healthcare sometimes helps. An even more inclusive concept is introduced at the very end of the article – ‘health practices’. If the meaning of this phrase is explained, it would often provide a good substitute for ‘healthcare'.

 p. 3, Introduction. The paper will exclude “professional migrants, marriage migrants, international students and tourists.” Firstly, this list should be harmonised with the list of exclusions given in Table 2 (“international students, international expatriates, tourists, healthcare professional migrants”). Tourists are in any case not normally classified as migrants, because they haven’t changed their place of usual residence. ‘Expatriates’ is a highly ambiguous term: it is colloquial rather than scientific, conveying the idea of being a cut above other migrants in some unspecified way. Migrants who have professional qualifications, and healthcare professionals in particular, can certainly find themselves placed in vulnerable, precarious or exploitative situations. For example, there is a large literature on the increased risks to which migrant workers (including health professionals) were exposed during the Covid-19 pandemic.  

 Secondly, the reasons for excluding these groups are not explained, except perhaps implicitly by defining the groups of interest as ‘vulnerable’ and/or ‘marginalised’. However, there are objections to labelling entire categories of migrants in this way. The aim is to further ‘levelling-up’ and remedy inequities, but such labelling inevitably involves a degree of stereotyping. In the body of the article two warnings are given about this:

- On p. 11, Khirikoekkong et al. (2020) are quoted as saying “labelling a study population as ‘vulnerable’ (such as ‘pregnant women’, ‘migrants’ or ‘children’) can potentially err in two ways: first, by unfairly excluding individuals who are capable of consenting or assenting (with some support) and depriving them of the potential benefits of research participation, and secondly, by not being protective enough of individuals who do not belong to designated vulnerable groups.”  
- The section on ‘Utility of Bioethics Research’ contains the following warning: “Using the concept of vulnerability as an example, migrants are considered to be vulnerable due to poverty or low socioeconomic status, precarity, poor access to healthcare, and discrimination. The use of such terms can be useful in describing the practical difficulties migrants face, but the widespread generalisation in the use of ‘vulnerability’ as a fixed descriptor for migrants without clear understanding of how they are or are not vulnerable may have potentially negative implications (Molenaar & Van Praag, 2022).” The rest of the paragraph describes very perceptively how being labelled as disadvantaged may often be a disadvantage in itself.

These warnings surely apply to the implicit assumption that professional migrants, marriage migrants and international students are not a suitable target group for an ethical approach. This is the only reason I can imagine for excluding them.

 p. 3, Migration: A Southeast Asian context. “Over the span of the last few decades, the number of migrants, has increased exponentially across the globe (McAuliffe & Triandafyllidou, 2021).”  The only reference to ‘exponential growth’ I could find in this publication was in connection with the increase in misinformation, of which this statement is a good example. ‘Exponential’ has a precise meaning, which is not applicable to the growth of migration (which actually slumped during the pandemic). Often the word is just a button that people press to create panic, which is not the atmosphere in which migration should be discussed.

 p. 4, Labour migration, “…..and *have* resulted in the region experiencing one of the highest rates of population mobility in the world”  (not *has *resulted).

 p. 5, top of page: “Migration from LMICs to high-income countries (HICs) forms the most prominent dialogue”.  It seems odd to call migration a ‘dialogue’: why not say something like “dominates current discussions”? This could be backed up with a reference to Wickramage et al. (2019).

 p. 5, Migration as a complex determinant of health. This section concludes “Migration is thus a complex determinant of health with dynamic interactions with legal, social, economic and public health considerations”. Couldn’t a sentence be inserted here adding *ethical* considerations to the above list?

 p. 6, Healthcare Access: see my comment on p. 1, Background. Title is too narrow, try e.g. “Ethics of health practices”

 p. 11, vulnerability: Freeman et al. (2021) did not say “only women experience pregnancy and childbirth, sexually transmitted infections (STIs)…and women are more likely to be subjected

 to sexual violence’. They wrote “Some sexually transmitted infections (STIs) may have a more severe impact on women than men”. The statement attributed to them is highly misleading.

 p. 16, Conclusion: “we conclude that bioethics scholarships have an undeniably crucial role……” Surely what was meant here was bioethics *scholarship, *not the provision of scholarships in bioethics?

 p. 16, Data availability: Appendix 1 is listed twice.

**My conclusions**

 The authors are to be congratulated on producing a fascinating and rich report, which goes some way to restoring the imbalance between LMICs and HICs in research and publications on migration health. I am happy to approve the paper, assuming that the authors will take note of the above comments and make the indicated minor changes where they feel this would improve the text.

 There remain some loose ends and contradictions in the paper, stemming from the fact that it is the first of its kind and that the authors’ views developed as the project proceeded. The place of studies on ‘health equity’ is a case in point: if the project were to be repeated, presumably the authors would not now want to exclude all epidemiological studies from it, just as they might be more reserved about labelling whole groups as marginalised, vulnerable or living in precarity.

Are the rationale for, and objectives of, the Systematic Review clearly stated?

Yes

Is the statistical analysis and its interpretation appropriate?

Not applicable

Are sufficient details of the methods and analysis provided to allow replication by others?

Yes

Are the conclusions drawn adequately supported by the results presented in the review?

Yes

Reviewer Expertise:

migrant inclusion in health systems, statistics on international migration

I confirm that I have read this submission and believe that I have an appropriate level of expertise to confirm that it is of an acceptable scientific standard.

# Response

**Published:** 2024-05-12

**Authors:** Shu Hui Ng

**Affiliations:** School of Medicine and Health Sciences, Monash University, Bandar Sunway, Selangor, Malaysia

***In response to Section 1 of the review, we have:***

***1)    Removed the ‘controversial 1948 definition of health’ as suggested.***

***2)    In the section of ‘Limitations’, we have changed that the terms are too ‘narrow’ instead of broad.***

***3)    For the sentence: “In the process of conducting this review, we found a significant number of epidemiological and socio-epidemiological studies that seek to identify differences in health and health care for different groups of migrants (Kim et al., 2023; Pham et al., 2019; Rajaraman et al., 2020)”, we have removed the references, and linked the sentence to Appendix 1.***

**Reviewer Comment:**

 p. 1: Background. This section mentions only healthcare and research – public health has been left out. All too many publications discuss ‘migrants and healthcare’, overlooking the fact that migrants also need to be included in health promotion and education, screening programmes, population research and health policy in general. In this article ‘healthcare’ is mentioned 37 times: the authors need to check that the other issues are not being neglected. Talking about health services rather than healthcare sometimes helps. An even more inclusive concept is introduced at the very end of the article – ‘health practices’. If the meaning of this phrase is explained, it would often provide a good substitute for ‘healthcare'.

***Author Response: Thank you. This paper looked into ‘healthcare’, which refers to efforts made to maintain, restore, or promote someone's physical, mental, or emotional well-being especially when performed by trained and licensed professionals. It is an overarching system of activities, institutions, and professionals involved in the maintenance and improvement of health, and encompasses medical treatment to health promotion and disease prevention, and covers a wide range of services and practices aimed at addressing individual and population health needs. We have added a footnote to the first sentence of ‘Introduction'.***

**Reviewer Comment:**

 p. 3, Introduction. The paper will exclude “professional migrants, marriage migrants, international students and tourists.” Firstly, this list should be harmonised with the list of exclusions given in Table 2 (“international students, international expatriates, tourists, healthcare professional migrants”). Tourists are in any case not normally classified as migrants, because they haven’t changed their place of usual residence. ‘Expatriates’ is a highly ambiguous term: it is colloquial rather than scientific, conveying the idea of being a cut above other migrants in some unspecified way. Migrants who have professional qualifications, and healthcare professionals in particular, can certainly find themselves placed in vulnerable, precarious or exploitative situations. For example, there is a large literature on the increased risks to which migrant workers (including health professionals) were exposed during the Covid-19 pandemic.  

 Secondly, the reasons for excluding these groups are not explained, except perhaps implicitly by defining the groups of interest as ‘vulnerable’ and/or ‘marginalised’. However, there are objections to labelling entire categories of migrants in this way. The aim is to further ‘levelling-up’ and remedy inequities, but such labelling inevitably involves a degree of stereotyping. In the body of the article two warnings are given about this:

 • On p. 11, Khirikoekkong et al. (2020) are quoted as saying “labelling a study population as ‘vulnerable’ (such as ‘pregnant women’, ‘migrants’ or ‘children’) can potentially err in two ways: first, by unfairly excluding individuals who are capable of consenting or assenting (with some support) and depriving them of the potential benefits of research participation, and secondly, by not being protective enough of individuals who do not belong to designated vulnerable groups.”  

 • The section on ‘Utility of Bioethics Research’ contains the following warning: “Using the concept of vulnerability as an example, migrants are considered to be vulnerable due to poverty or low socioeconomic status, precarity, poor access to healthcare, and discrimination. The use of such terms can be useful in describing the practical difficulties migrants face, but the widespread generalisation in the use of ‘vulnerability’ as a fixed descriptor for migrants without clear understanding of how they are or are not vulnerable may have potentially negative implications (Molenaar & Van Praag, 2022).” The rest of the paragraph describes very perceptively how being labelled as disadvantaged may often be a disadvantage in itself.

 These warnings surely apply to the implicit assumption that professional migrants, marriage migrants and international students are not a suitable target group for an ethical approach. This is the only reason I can imagine for excluding them.

***Author Response: We have harmonised the text in Introduction and Table 2. Professional migrants, marriage migrants, international students and tourists are not populations in which the experience of migration itself creates significant risks and/or barriers to health. It should be acknowledged however that members of these populations can find themselves placed in vulnerable and precarious situations for their health and healthcare. We did not label these populations as ‘vulnerable’ or ‘not vulnerable’, and they should also be included in an ethical approach to healthcare, albeit outside of the scope of this paper.***

**Reviewer Comment:**

 p. 3, Migration: A Southeast Asian context. “Over the span of the last few decades, the number of migrants, has increased exponentially across the globe (McAuliffe & Triandafyllidou, 2021).”  The only reference to ‘exponential growth’ I could find in this publication was in connection with the increase in misinformation, of which this statement is a good example. ‘Exponential’ has a precise meaning, which is not applicable to the growth of migration (which actually slumped during the pandemic). Often the word is just a button that people press to create panic, which is not the atmosphere in which migration should be discussed.

***Author Response: The word ‘exponential’ has been deleted.***

**Reviewer Comment:**

 p. 4, Labour migration, “…..and have resulted in the region experiencing one of the highest rates of population mobility in the world”  (not has resulted).

***Author Response:*** ***Thank you, corrected.***

**Reviewer Comment:**

 p. 5, top of page: “Migration from LMICs to high-income countries (HICs) forms the most prominent dialogue”.  It seems odd to call migration a ‘dialogue’: why not say something like “dominates current discussions”? This could be backed up with a reference to Wickramage et al. (2019).

***Author Response:*** ***Edited per suggestion.***

**Reviewer Comment:**

 p. 5, Migration as a complex determinant of health. This section concludes “Migration is thus a complex determinant of health with dynamic interactions with legal, social, economic and public health considerations”. Couldn’t a sentence be inserted here adding ethical considerations to the above list?

***Author Response:*** ***Added ‘ethical’ per suggestion.***

 p. 6, Healthcare Access: see my comment on p. 1, Background. Title is too narrow, try e.g. “Ethics of health practices”

***Please see response to comment  p.1.***

**Reviewer Comment:**

 p. 11, vulnerability: Freeman et al. (2021) did not say “only women experience pregnancy and childbirth, sexually transmitted infections (STIs)…and women are more likely to be subjected to sexual violence’. They wrote “Some sexually transmitted infections (STIs) may have a more severe impact on women than men”. The statement attributed to them is highly misleading. 

***Author Response: Sentence corrected.***

**Reviewer Comment:**

 p. 16, Conclusion: “we conclude that bioethics scholarships have an undeniably crucial role……” Surely what was meant here was bioethics scholarship, not the provision of scholarships in bioethics?

***Author Response:*** ***Edited to 'bioethics scholarship'.***

**Reviewer Comment:**

 p. 16, Data availability: Appendix 1 is listed twice .

***Author Response: We have double checked and it should be listed only once in our manuscript.*** ***Thank you very much for your time and constructive feedback!***

# Reviewer report: Reviewer response for version 2

**DOI:** 10.21956/wellcomeopenres.22412.r70286

**Published:** 2023-12-16

**Authors:** Roomi Aziz

**Affiliations:** 1 School of Health and Social Care, University of Essex, Colchester, England, UK

Dear Authors,

 Thank you for addressing all the comments and taking the feedback kindly.

 I do feel that this reads much better. The conclusion is much more crisp and to-the-point for a lay reader, helping them in understanding the gist of the study.

 Just two final comments:

 1. I still think that the authors need to briefly describe the analysis process. Once the 18 articles were scoped and charted, how have you analysed and extracted the ethical concepts and the research ethics presented in the results section. Have you extracted themes? I understand that the modality was not decided at the outset, but now that the analysis has been done, what guidelines or references were followed for this kind of analysis. This would make the method more replicable and help the reader in understanding your process.

 2. The closing paragraph comments on the need of a research agenda where ethics is centered. Now that WHO's migration and health global research agenda is out, can a reference to the same be made, along with a brief comment on how central does ethics seem to the agenda, or reiterating the need of regional expertise after having a look at the research agenda.

 Overall thanking you for considering my comments on this review.

Are the rationale for, and objectives of, the Systematic Review clearly stated?

Yes

Is the statistical analysis and its interpretation appropriate?

Not applicable

Are sufficient details of the methods and analysis provided to allow replication by others?

Partly

Are the conclusions drawn adequately supported by the results presented in the review?

Partly

Reviewer Expertise:

Migration health, Human resources for health, Systematic and Scoping Reviews, Health Systems

I confirm that I have read this submission and believe that I have an appropriate level of expertise to confirm that it is of an acceptable scientific standard.

# Reviewer report: Reviewer response for version 1

**DOI:** 10.21956/wellcomeopenres.21682.r67505

**Published:** 2023-10-10

**Authors:** Roomi Aziz

**Affiliations:** 1 School of Health and Social Care, University of Essex, Colchester, England, UK

First of all, I would like to take this opportunity to thank the authors and the journal for inviting me to review this submission.

 Overall, the scoping review intends to map key themes around ethics of healthcare for, and health research involving marginalised migrant populations in South-East Asia. I enjoyed reading it and have provided my comments below.

 It is good that the authors have highlighted the scope of the review in the beginning, so that the reader knows what to expect vis-à-vis migrant population groups excluded from the review’s scope.

 Footnote 2 may actually be included in the main text as it is valuable for the reader to note key numbers like 281 million international and at least 740 million internal migrants i.e. the volume of people on the move in the world.

 I do feel there is extensive discussion around migration as determinant of health and would rather prefer that the authors set the context and then move towards the scoping review itself.

 Since Table 1 is not entirely exhaustive, this can be removed.

 Table 2 could be transformed into a visual and used to strengthen the discussion after the results are presented, as opposed to being placed where they are in the article at present.

 Overall, the content under ‘Migration as a complex determinant of health’ i.e. from Pg5 to Pg7 could be reduced, better organized and made crisper, with clearer signposting. Right now, this section begins with defining health, explaining migration health vs. migrant health, policy-responsiveness to migrant health needs, precarious employment, again moving to relationship with the state, trafficking, xenophobia, SDGs’ acknowledgement. Some of these components have already been elaborated in the key drivers of migration discussed earlier.

 Since it has been clearly defined in the beginning that the review concerns ethics around access to health, as well as research, it would fare better if the section ‘’The role of bioethics in SEA migration health’ clearly and explicitly explains both components in more depth.

 My comments specific to the scoping review are listed below:

1. If the search strategy presented in Table 3 is the final search strategy, I do feel that this is limiting, and does not cover a large number of migrant population keywords which otherwise have not been excluded from the scope. E.g. 'urban-rural movement', 'labor migrants', 'gypsies', 'exiles', 'uprooted', 'temporary', 'irregular', 'illegal', 'undocumented', 'forced labour' etc. to name a few (see Sweileh's search strategy ^1^). Similarly for health, keywords like 'well-being', 'illness', 'disease', 'disorders'. For ethics, this would be 'research integrity', 'research misconduct', 'human rights', 'rights-based', 'consent', 'confidential', 'best practices', 'moral' etc. However at this point, a practical step would be to elaborate the limitations of the search string and selected keywords, as well as the rationale behind the same.
2. It would help the users if the complete search string is provided as a supplementary file, also indicating if this search string was run for Title, or Abstract, or Title-Abstract-Keyword.
3. If migrant populations are under consideration, databases like SCOPUS would add more value compared to CINAHL. Alternatively, rationale behind choosing the final databases may be provided to strengthen the authors’ case.
4. Can the authors also provide the reason behind restricting search to 2000 and beyond.
5. The authors can edit out their comments on not doing critical appraisal, since critical appraisal of sources included is completely optional in a scoping review, and only needs to be reported if it was done (See JBI and PRISMA Guidelines for Scoping review reporting ^2^). Subsequently this limitation can be removed from Pg17.
6. In the results section, please describe how this thematic/ conceptual analysis was undertaken i.e. was this analysis modality decided at the outset? References or rationale for the same may also be added.
7. The beginning of the results section would benefit from some content analysis of the final selected 18 studies (which is all listed in Table 4 but would give an overview of the included studies i.e. 50% of the included studies were set in Thailand (n=9), 7 in Singapore and 1 in Malaysia. Maybe then some commentary on the missing countries, especially Philippines, Myanmar and Indonesia. Similarly, types of studies etc., types of study population i.e. children or adults, internal or international etc. This is specifically important since later in the Research Gaps, the authors have highlighted evidence paucity around migrant children as well as internal migrants.
8. Overall I found the results sections a bit too long and monotonous in places. Perhaps a little more brevity/condensing of the content would help.
9. In the discussion section (or the conclusion section), A) as a Migration Health researcher, what would really benefit me is a list of 5-10 things to do, to address any potential ethical issues one could experience during designing and conducting research on migration health, ethical considerations and applications, and things to do in ethically challenging circumstances. B) A similar list for medical practitioners in clinical settings. C) As a researcher interested in ethics, key areas that I should be exploring to study ethics in migration health. Something like key take-aways. That would really set this review apart.
10. Can more details be provided for the regional stakeholders’ consultation mentioned on Pg16 (under Research Gaps identified).
11. Suggesting that if PAR is being identified as a research gap, then the results section should also comment on types of research to make the connection easier for readers to follow.
12. Most of the research gaps identified refer to subjects/methods unexplored e.g. migrant children, internal migrants, migrant-sending countries, PAR, there is little spoken about key gaps in ethics around MH research and healthcare access. A suggestion would be to re-label this to ‘Other research gaps’, since the discussion section does talk about utilising bioethics research.
13. Can the note on limitations be expanded, to emphasize that the broad search terms used have impacted the size and composition of the outcome of the review here, and that the research gaps listed in this review should be viewed with this consideration.

Are the rationale for, and objectives of, the Systematic Review clearly stated?

Yes

Is the statistical analysis and its interpretation appropriate?

Not applicable

Are sufficient details of the methods and analysis provided to allow replication by others?

Partly

Are the conclusions drawn adequately supported by the results presented in the review?

Partly

Reviewer Expertise:

Migration health, Human resources for health, Systematic and Scoping Reviews, Health Systems

I confirm that I have read this submission and believe that I have an appropriate level of expertise to confirm that it is of an acceptable scientific standard, however I have significant reservations, as outlined above.

## References

[1] : Bibliometric analysis of global migration health research in peer-reviewed literature (2000-2016). *BMC Public Health* .2018;18(1) : 10.1186/s12889-018-5689-x 777 10.1186/s12889-018-5689-x 29925353 PMC6011263

[2] : PRISMA Extension for Scoping Reviews (PRISMA-ScR): Checklist and Explanation. *Ann Intern Med* .2018;169(7) : 10.7326/M18-0850 467-473 10.7326/M18-0850 30178033

# Response

**Published:** 2023-11-08

**Authors:** Shu Hui Ng

**Affiliations:** School of Medicine and Health Sciences, Monash University, Bandar Sunway, Selangor, Malaysia

First of all, I would like to take this opportunity to thank the authors and the journal for inviting me to review this submission.Overall, the scoping review intends to map key themes around ethics of healthcare for, and health research involving marginalised migrant populations in South-East Asia. I enjoyed reading it and have provided my comments below. It is good that the authors have highlighted the scope of the review in the beginning, so that the reader knows what to expect vis-à-vis migrant population groups excluded from the review’s scope.

*Thank you for your time reviewing this review and your valuable comments. Please see our responses below. *  

1. Footnote 2 may actually be included in the main text as it is valuable for the reader to note key numbers like 281 million international and at least 740 million internal migrants i.e. the volume of people on the move in the world.

*Thank you for this suggestion, we have incorporated the content of footnote 2 into the text. *

1. I do feel there is extensive discussion around migration as determinant of health and would rather prefer that the authors set the context and then move towards the scoping review itself.

*The elaboration and evidence on how migration impacts migrants’ and their families’ health after a summarising table (originally Table 2) has been removed. *

1. Since Table 1 is not entirely exhaustive, this can be removed.

*Table 1 is removed as suggested. *

1. Table 2 could be transformed into a visual and used to strengthen the discussion after the results are presented, as opposed to being placed where they are in the article at present.

*As we would like to focus on the outcome of the review in the discussion, Table 2 (now Table 1) is maintained in its original section. *

1. Overall, the content under ‘Migration as a complex determinant of health’ i.e. from Pg5 to Pg7 could be reduced, better organized and made crisper, with clearer signposting. Right now, this section begins with defining health, explaining migration health vs. migrant health, policy-responsiveness to migrant health needs, precarious employment, again moving to relationship with the state, trafficking, xenophobia, SDGs’ acknowledgement. Some of these components have already been elaborated in the key drivers of migration discussed earlier.

*Thank you for highlighting this, edited per response at point 2. *

1. Since it has been clearly defined in the beginning that the review concerns ethics around access to health, as well as research, it would fare better if the section ‘’The role of bioethics in SEA migration health’ clearly and explicitly explains both components in more depth.
*We have made some edits to the section with subheadings to delineate both components. *

My comments specific to the scoping review are listed below:

1. If the search strategy presented in Table 3 is the final search strategy, I do feel that this is limiting, and does not cover a large number of migrant population keywords which otherwise have not been excluded from the scope. E.g. 'urban-rural movement', 'labor migrants', 'gypsies', 'exiles', 'uprooted', 'temporary', 'irregular', 'illegal', 'undocumented', 'forced labour' etc. to name a few (see Sweileh's search strategy ^1^ ). Similarly for health, keywords like 'well-being', 'illness', 'disease', 'disorders'. For ethics, this would be 'research integrity', 'research misconduct', 'human rights', 'rights-based', 'consent', 'confidential', 'best practices', 'moral' etc. However at this point, a practical step would be to elaborate the limitations of the search string and selected keywords, as well as the rationale behind the same.

*The keywords are selected as the final search strategy because multiple trials with different combinations of keywords yielded an extensive list of publications of limited bioethics relevance. The limitation of the final search strategy was elaborated under the section ‘limitations’.*

1. It would help the users if the complete search string is provided as a supplementary file, also indicating if this search string was run for Title, or Abstract, or Title-Abstract-Keyword.

*The section on the search strategy has been edited. A separate supplementary file containing the search string has been provided. *

1. If migrant populations are under consideration, databases like SCOPUS would add more value compared to CINAHL. Alternatively, rationale behind choosing the final databases may be provided to strengthen the authors’ case.
* *
*Please see our revised ‘search strategy’ section. The Cumulative Index of Nursing and Allied Health Literature (CINAHL) was included to capture potential migrant healthcare and related ethical issues from nursing and allied health perspectives, as no single database could capture all (or most) bioethics research. *
2. Can the authors also provide the reason behind restricting search to 2000 and beyond.

*Bioethics is a developing field in Southeast Asia. Year 2000 and beyond is selected to reflect current bioethics discourse in the region.  *

1. The authors can edit out their comments on not doing critical appraisal, since critical appraisal of sources included is completely optional in a scoping review, and only needs to be reported if it was done (See JBI and PRISMA Guidelines for Scoping review reporting ^2^ ). Subsequently this limitation can be removed from Pg17.

*Comments on not doing a critical appraisal in both sections (Analysis and Limitations) have been removed.  *

1. In the results section, please describe how this thematic/ conceptual analysis was undertaken i.e. was this analysis modality decided at the outset? References or rationale for the same may also be added.

*We did not decide on the analysis modality at the outset because we were not sure what bioethics research was available.*

1. The beginning of the results section would benefit from some content analysis of the final selected 18 studies (which is all listed in Table 4 but would give an overview of the included studies i.e. 50% of the included studies were set in Thailand (n=9), 7 in Singapore and 1 in Malaysia. Maybe then some commentary on the missing countries, especially Philippines, Myanmar and Indonesia. Similarly, types of studies etc., types of study population i.e. children or adults, internal or international etc. This is specifically important since later in the Research Gaps, the authors have highlighted evidence paucity around migrant children as well as internal migrants.

*Thank you for the valuable suggestions. The beginning of the results section has been edited per suggestions. *

1. Overall I found the results sections a bit too long and monotonous in places. Perhaps a little more brevity/condensing of the content would help.

*We have further summarised the results sections.*

1. In the discussion section (or the conclusion section), A) as a Migration Health researcher, what would really benefit me is a list of 5-10 things to do, to address any potential ethical issues one could experience during designing and conducting research on migration health, ethical considerations and applications, and things to do in ethically challenging circumstances. B) A similar list for medical practitioners in clinical settings. C) As a researcher interested in ethics, key areas that I should be exploring to study ethics in migration health. Something like key take-aways. That would really set this review apart.
*We have edited the conclusion section, citing Onarheim et al. *
2. Can more details be provided for the regional stakeholders’ consultation mentioned on Pg16 (under Research Gaps identified).

*Regional stakeholders, including migrant health researchers, members of non-governmental organization and clinicians, were engaged in informal online conversations prior to and during the scoping review. These discussions involved open-ended questions on their perspectives on gaps in migrant health research without a formal interview guide or protocol.*

1. Suggesting that if PAR is being identified as a research gap, then the results section should also comment on types of research to make the connection easier for readers to follow.

*From the retrieved articles, only studies from Thailand adopted a participatory research approach, characterised by greater and more direct involvement of migrants in research processes, through the formation of a Community Advisory Board (CAB).*

1. Most of the research gaps identified refer to subjects/methods unexplored e.g. migrant children, internal migrants, migrant-sending countries, PAR, there is little spoken about key gaps in ethics around MH research and healthcare access. A suggestion would be to re-label this to ‘Other research gaps’, since the discussion section does talk about utilising bioethics research.
*Relabelled to ‘Other research gaps’. *
2. Can the note on limitations be expanded, to emphasize that the broad search terms used have impacted the size and composition of the outcome of the review here, and that the research gaps listed in this review should be viewed with this consideration.

*The section on limitation has been edited. * Thank you very much once again.

# Reviewer report: Reviewer response for version 1

**DOI:** 10.21956/wellcomeopenres.21682.r66711

**Published:** 2023-09-26

**Authors:** Ornella Punzo

**Affiliations:** 1 Istituto Superiore di Sanità, Roma, Italy

This scoping review aims to analyse key themes and gaps in research regarding the ethics of migrant health and research ethics in this field in Southeast Asia. I found the paper interesting, although some parts might be a bit long and repetitive.

 The rationale and objectives are clearly stated. Sufficient details of the methods and analysis are provided. The conclusions drawn are adequately supported by the results presented in the review.

 I have some questions and remarks on the methods.

 First, why did the authors choose these three literature databases? While usually librarians suggest that Ovid is a more appropriate option than Pubmed, I do not understand the choice of CINAHL for this specific research question involving ethics and research ethics, especially given that only three databases were interrogated.

 A critical appraisal of the papers retrieved was not performed; in the methods section, the authors say that this was a choice as they aimed at mapping existing literature and identifying key concepts and gaps, more than at synthesising these works. However, in the limitations paragraph, they admit there was a lack of capacity to perform quality appraisals of publications.

 In the search strategy, in the inclusion criteria section, there is no mention of study designs. In the table with the included studies’ characteristics, there is a column regarding the study designs. I understand that, given the subject, it would be more difficult to retrieve non-qualitative studies, but I find it difficult to justify a lack of attention to the type of studies the authors intended to include from the start. This issue is linked to the previous point too, as there are also three commentaries included in the scoping review, and not having a critical appraisal or a strict inclusion criterion based on the study design seems relevant.

 Even if there is an effort to explain what bioethics is and what its value would be in the field of migrant health and its research, I sometimes find the connections between ethics and social determinants of health, including migrant health, a bit vague. I would expect a bit more and also in other sections, besides what the authors write on page 5 regarding socioeconomic factors and their links to migrant health and migrant health research.

Are the rationale for, and objectives of, the Systematic Review clearly stated?

Yes

Is the statistical analysis and its interpretation appropriate?

Not applicable

Are sufficient details of the methods and analysis provided to allow replication by others?

Yes

Are the conclusions drawn adequately supported by the results presented in the review?

Yes

Reviewer Expertise:

Migrant health, Infectious diseases epidemiology, Climate change and health

I confirm that I have read this submission and believe that I have an appropriate level of expertise to confirm that it is of an acceptable scientific standard, however I have significant reservations, as outlined above.

# Response

**Published:** 2023-11-08

**Authors:** Shu Hui Ng

**Affiliations:** School of Medicine and Health Sciences, Monash University, Bandar Sunway, Selangor, Malaysia

This scoping review aims to analyse key themes and gaps in research regarding the ethics of migrant health and research ethics in this field in Southeast Asia. I found the paper interesting, although some parts might be a bit long and repetitive. The rationale and objectives are clearly stated. Sufficient details of the methods and analysis are provided. The conclusions drawn are adequately supported by the results presented in the review.  

**Thank you for your time in reviewing this paper. We appreciate all your questions and suggestions. Please find our responses below.**

 I have some questions and remarks on the methods.  

1. First, why did the authors choose these three literature databases? While usually librarians suggest that Ovid is a more appropriate option than Pubmed, I do not understand the choice of CINAHL for this specific research question involving ethics and research ethics, especially given that only three databases were interrogated.

**The Cumulative Index of Nursing and Allied Health Literature (CINAHL) was included to capture potential migrant healthcare and related ethical issues from nursing and allied health perspectives, as no single database could capture all (or most) bioethics research.  **

1. A critical appraisal of the papers retrieved was not performed; in the methods section, the authors say that this was a choice as they aimed at mapping existing literature and identifying key concepts and gaps, more than at synthesising these works. However, in the limitations paragraph, they admit there was a lack of capacity to perform quality appraisals of publications.

**In consideration of our second reviewer’s comment (#11), we have removed the sentence on ‘mapping existing literature’ and revised the ‘limitations’ paragraph accordingly. For scoping reviews, there is no absolute need for critical appraisal.  **

1. In the search strategy, in the inclusion criteria section, there is no mention of study designs. In the table with the included studies’ characteristics, there is a column regarding the study designs. I understand that, given the subject, it would be more difficult to retrieve non-qualitative studies, but I find it difficult to justify a lack of attention to the type of studies the authors intended to include from the start. This issue is linked to the previous point too, as there are also three commentaries included in the scoping review, and not having a critical appraisal or a strict inclusion criterion based on the study design seems relevant.

**Thank you for this observation. The aim of this research was to find out what bioethics research we could find and what types of research were done in this region. The types of research or publications are the results of the scoping review.  **

1. Even if there is an effort to explain what bioethics is and what its value would be in the field of migrant health and its research, I sometimes find the connections between ethics and social determinants of health, including migrant health, a bit vague. I would expect a bit more and also in other sections, besides what the authors write on page 5 regarding socioeconomic factors and their links to migrant health and migrant health research.

**Based on what we found through this research, we agree with the reviewer that the connections between ethics and social determinants of health are indeed vague. We see this as one value of the paper, which points to the need for more research and analysis to make evident the value of bioethics to migration health, and exactly how ethical values and practices can (and should) implicate migrants’ social determinants of health.  Thank you very much once again.**
